# Leucocythæmia

**Published:** 1852-08

**Authors:** 


*Leucocythæmia.*—Prof. G. B. Wood called the attention of the college to a form of disease described by Dr. Bennett, of Edinburgh, under this name, the leading characteristic of which is an excess of the white corpuscles of the blood. He stated that there was a case of it in the Pennsylvania Hospital. The patient, a male of about seventeen years of age, was admitted laboring under symptoms of anæmia, with some anasarcous effusion, general debility, and great enlargement of the spleen. The blood was examined by Dr. Adenell Hewson, resident physician of the hospital, who is accustomed to microscopic investigations, and found by him to contain a great excess of white corpuscles. There could be no doubt that it was a case of the leucocythæmia of Dr. Bennett. The patient was put upon the use of iron and quinia, with a good diet. Under this treatment, the dropsical symptoms and enlargement of the spleen rapidly diminished, and the patient soon became restored to a degree of robustness, which was in strong contrast with the debilitated appearance he presented at his entrance into the institution. The spleen was evidently diminished in bulk; but at the same time the liver was found to have become enlarged. On examining the blood under the microscope, it still exhibited the same excess of white corpuscles. Dr. Wood supposed that the patient had been overstimulated by the treatment to which he had been subjected. A less invigorating diet was directed, small doses of blue mass were administered, and a blister was applied over the right hypochondriac region. Under this treatment the condition of the liver became improved; but, as the symptoms of anæmia reappeared, and the spleen began again to enlarge, a blister was applied over the left hypochondrium, and nitro-muriatic acid was substituted for mercury. The visceral disease now rapidly diminished; but as the anæmia continued, recourse was again had to chalybeates. All the symptoms now improved; and an examination of the blood showed a very considerable diminution in the number of white corpuscles. When the patient was last seen by Dr. Wood, the spleen was of nearly its natural dimensions, and the anæmic symptoms had almost disappeared; but the liver still remained somewhat enlarged.—*Quart. Sum. Trans. Coll. Phys. Philadelphia, Jan. to April,* 1852.

